# AMPK Signaling Pathway as a Potential Therapeutic Target for Parkinson’s Disease

**DOI:** 10.34172/apb.2024.013

**Published:** 2023-10-14

**Authors:** Seyed Zanyar Athari, Fereshteh Farajdokht, Rana Keyhanmanesh, Gisou Mohaddes

**Affiliations:** ^1^Drug Applied Research Center, Tabriz University of Medical Sciences, Tabriz, Iran.; ^2^Neurosciences Research Center, Tabriz University of Medical Sciences, Tabriz, Iran.; ^3^Department of Biomedical Education, California Health Sciences University, College of Osteopathic Medicine, Clovis, CA, USA.

**Keywords:** Parkinson's disease, AMPK, α-Synuclein, Oxidative stress, Inflammation

## Abstract

Parkinson’s disease (PD) is the second most common neurodegenerative disease caused by the loss of dopaminergic neurons. Genetic factors, inflammatory responses, oxidative stress, metabolic disorders, cytotoxic factors, and mitochondrial dysfunction are all involved in neuronal death in neurodegenerative diseases. The risk of PD can be higher in aging individuals due to decreased mitochondrial function, energy metabolism, and AMP-activated protein kinase (AMPK) function. The potential of AMPK to regulate neurodegenerative disorders lies in its ability to enhance antioxidant capacity, reduce oxidative stress, improve mitochondrial function, decrease mitophagy and macroautophagy, and inhibit inflammation. In addition, it has been shown that modulating the catalytic activity of AMPK can protect the nervous system. This article reviews the mechanisms by which AMPK activation can modulate PD.

## Introduction

 Parkinson’s disease (PD) is the second most common neurodegenerative disease caused by the loss of dopaminergic neurons.^1-3^ In 2016, it was estimated that PD affected 6.1 million people worldwide, up from 2.5 million in 1990, and this figure is predicted to more than double by 2040.^4^ Moreover, PD is present in approximately 3% of individuals aged 65 and above, with the largest number of cases reported in those over 70 years old.^5^

 Clinically, symptoms of PD can be categorized as non-motor signs and motor symptoms. The non-motor symptoms are more common and emerge years before motor symptoms.^6^ Non-motor symptoms comprise loss of sense of smell, sensory disturbances (such as pain), sleep disorders, autonomic disorders (orthostatic hypotension), gastrointestinal disorders (constipation), urogenital disorders, sexual dysfunction, as well as cognitive deficits and dementia.^7^ Motor symptoms include bradykinesia, tremors at rest, rigid muscles, impaired posture, and imbalance. In addition to the main symptoms, patients may show other motor symptoms like micrography, freezing, masked face, decreased blink rate, dysphagia, and softened voice.^8^

 Pathologically, the key characteristics of PD are the damage to dopaminergic neurons in substantia nigra pars compacta (SNpc) and ventral tegmental area (VTA), depletion of dopamine in the striatum, and the presence of Lewy bodies in the cytoplasm formed mainly by the α-synuclein (α-syn) protein. PD affects various neurotransmitters aside from the dopamine system, such as noradrenaline, serotonin, glutamate, γ-aminobutyric acid (GABA), acetylcholine, and neuropeptides. The development of PD may also be caused by the degeneration of cholinergic neurons in the meynert nucleus, norepinephrinergic neurons in the locus cereus, and serotoninergic neurons in the raphe nuclei.^9^ Non-motor symptoms caused by non-dopaminergic neurotransmitter system dysfunction are unresponsive to dopaminergic therapy.^10^

 Along with genetic factors, inflammation, oxidative stress, mitochondrial dysfunction, and cytotoxic factors,^11,12^ metabolism-related dysfunction is also involved in the pathophysiology of PD.^13^ Evidence shows that impaired regulation of glucose metabolism, which occurs in early PD, reduces antioxidant capacity and neuronal survival.^14^ Furthermore, during the initial stages of PD, oxidative stress, a crucial characteristic of metabolic syndrome, leads to mitochondrial structural abnormalities and mutations in mitochondrial DNA, which worsen oxidative stress and ultimately cause neuronal death.^15^

 Energy dysregulation is implicated as a possible trigger for PD, indicating that a deeper understanding of the molecular pathways controlling energy balance could lead to identifying therapeutic targets. The AMP-activated protein kinase (AMPK) signaling pathway regulates metabolism, cell growth, and autophagy,^16^ and serves as a metabolic energy sensor and controls both lipid and carbohydrate metabolism inside the cell.^17,18^ Moreover, inhibiting AMPK expression or activity results in an increase in pro-inflammatory cytokines such as interleukin (IL)-1, IL-6, and tumor necrosis factor (TNF)-α,^19^ whereas stimulating AMPK pathway has been shown to boost neuroprotection.^20^ AMPK is also involved in regulating macroautophagy,^21^ mitochondrial biogenesis,^22^ and gene expression.^23^

 Energy balance in cells is maintained by AMPK, which inhibits energy consumption and activates energy production processes in response to specific conditions to restore ATP levels.^24^ The mitochondrial oxidative-phosphorylation (OxPh) pathway is commonly used to produce ATP from glucose. Hence, increasing AMPK activity is a viable strategy to avoid bioenergetics failure and boost energy levels in vulnerable neurons.^25^ AMPK stimulates glucose transport through glucose membrane vectors and the breakdown of stored glycogen in the cytoplasm.^25,26^ AMPK also provides substrates for other OxPh sources like fatty acids (FA) and glutamine.^27^ During calorie restriction, AMPK acutely increases the uptake and transfer of FAs to the mitochondria for catabolism, oxidation, and energy production. Long-term activation of AMPK can influence energy metabolism by activating regulatory factors like forkhead box transcription factors (FOXO) and peroxisomal proliferator-activated receptor-gamma coactivator (PGC)-1 α for energy production and consumption.^28-30^

 Furthermore, AMPK regulates cellular ATP production and energy levels by restricting anabolic processes.^24^ AMPK inhibits processes that require ATP, like new protein production and cell growth, to maintain the ATP level in energy-constrained conditions.^31^ The mammalian target of rapamycin complex (mTORC)-1 is an essential cellular protein that promotes protein synthesis and growth and induces nutrient signals.^24^ Evidence shows that AMPK inhibits mTORC-1 through activating tuberous sclerosis complex (TSC)-2 and inhibiting regulatory-associated protein of mTOR (RAPTOR).^32,33^ Also, AMPK has the ability to decrease protein production by inhibiting the synthesis of ribosomal RNA.^34^

## AMPK effect on mitochondrial function

 Cell metabolism relies on organelles called mitochondria, which provide energy through the OxPh process. The OxPh generates additional substances, particularly reactive oxygen species (ROS), that can negatively affect mitochondrial function when produced excessively. The decrease in cellular energy production following mitochondrial dysfunction creates a vicious cycle of chronic ROS production and worsens mitochondrial dysfunction.^35^ Therefore, cells’ essential functions are to control mitochondrial health, biogenesis, fission-fusion dynamics, and mitochondrial autophagy (mitophagy).^36^ The process of mitochondrial quality control declines with age, particularly in those with PD.^37,38^ Additionally, accumulated α-syn may be the reason for mitochondrial damage in PD.^39,40^

 Mitochondria can change their structure, size, and shape through repetitive cycles of fission and fusion.^41^ Mitochondrial dynamics can be influenced by calcium homeostasis, apoptosis, and respiration. Genetic mutations or exposure to toxins can lead to changes in mitochondrial dynamics, causing neurodegenerative disorders. The fusion of mitochondria is accomplished by two groups of GTPases: mitofusins (MFN1 and MFN2) located in the outer mitochondrial membrane and optic atrophy (OPA)-1 located in the inner mitochondrial membrane.^42^ Fission is another alteration in mitochondrial dynamics where dynamin-related protein (DRP)-1 is the key factor.^43^

 Dopaminergic neurons in the SNpc have limited mitochondrial content and rely heavily on energy balance for survival.^44^ Sporadic and familial forms of PD affect diverse aspects of mitochondria, including their bioenergy capacity, quality control, life cycle, morphology (fission and fusion), transportation, and control of cellular apoptosis pathways.^45^ Furthermore, *PINK1* and *PARKIN* genes play a key role in mitochondrial function and quality control as they detect damage in mitochondria and facilitate mitophagy to eliminate and replace dysfunctional mitochondrial components.^46,47^ Ubiquitination of MFN1 and MFN2 proteins, which are involved in mitochondria fusion, depends on the Parkin/PINK1 pathway, wherein PINK1 phosphorylates MFN2, resulting in Parkin recruitment and protein ubiquitination.^48^ This process is essential to identify mitochondria for degradation through mitophagy and prevent them from reintegrating into the mitochondrial network. However, this process is disturbed by PD, leading to the accumulation of abnormal mitochondria and respiratory dysfunction. Moreover, loss of DRP-1 in dopaminergic neurons leads to the degeneration of SN neurons in mice and a Parkinson’s-like phenotype due to depletion of axonal mitochondria.^49^

 One of the primary regulators of mitochondrial biogenesis is a transcriptional activator called PGC-1.^50^ According to prior studies, PD causes a decline in the expression of PGC-1 and its downstream genes responsible for controlling cellular bioenergy and mitochondrial biogenesis.^51,52^ Interestingly, overexpression of PGC-1 can prevent dopaminergic neuron death caused by α-syn overexpression or rotenone-induced damage, potentially improving PD-like pathologies.^52^

 As AMPK is vital for intracellular energy metabolism in response to energy depletion, it is expected that AMPK has a significant impact on mitochondrial homeostasis. An in vitro study has shown that α-syn overexpression reduces AMPK activity, leading to a decrease in cellular resistance to α-syn.^53^ A deficiency in AMPK activity can lead to reduced mitochondria and abnormal mitochondrial biogenesis due to disruption of the AMPK/PGC-1 axis, putting dopaminergic neurons at risk of degeneration and causing symptoms similar to PD.^54,55^ However, pharmacological AMPK activation provides neuroprotection.^55^

 Through activating PGC-1α, AMPK promotes mitochondrial biogenesis, activating mitochondrial transcription factor A (TFAM), leading to increased transcription and replication of mitochondrial DNAs.^56,57^ Furthermore, AMPK enhances mitochondrial fusion, leading to the development of extensive and highly branched mitochondrial networks in a PGC-1-dependent way.^58,59^ Besides, AMPK activates mitochondrial fission factor (MFF) to promote mitochondrial fission but inhibits mTORC1 to suppress it.^60,61^ Therefore, it seems that the role of AMPK in intervening mitochondrial homeostasis is context-dependent based on cellular energy status. In mild energy depletion, it may stimulate fusion to boost energy production, but under prolonged and intense cellular stress, it may trigger fission to promote mitophagy and initiate mitochondrial biogenesis to substitute the impaired ones.

 AMPK also facilitates mitochondrial function by controlling the direct phosphorylation of target proteins and transcriptional regulation of the relevant genes.^62^ Mitophagy is a physiological process that eliminates damaged mitochondria while promoting mitochondrial biogenesis pathways to replenish mitochondrial levels.^63,64^ Through the phosphorylation of Unc-51-like autophagy activating kinase (ULK)-1, AMPK promotes mitophagy by facilitating autophagosome formation and directing damaged mitochondria to lysosomes.^65^ AMPK activation also couples mitochondria fission with mitophagy by phosphorylating MFF and activating DRP-1 to maintain energy bioavailability and high-quality mitochondria.^66,67^

 The mitochondrial electron transport chain is the major source of ROS, and cells rely on antioxidant mechanisms to prevent damage from ROS and maintain redox homeostasis. Proper cellular function and metabolic stress adaptation necessitate the regulation of ROS generated by mitochondria.^68,69^ Damage to essential cellular components caused by excessive free radical production and impaired redox balance in neurons contributes to the degeneration of dopaminergic neurons in the SN. The low glutathione levels, high levels of oligomeric α-syn, high iron and calcium contents, mitochondrial dysfunction, and dopamine degradation and oxidation are responsible for ROS production in PD.^70,71^ Genetic mutations in *SNCA*, *PARKIN*, *PINK1*, *LRRK2*, *FBXO7*, *ATP13A2*, *GIGYF2* and *HTRA2* are also responsible for impairing mitochondrial function and morphology, leading to ROS formation.^72^ The connection between oxidative stress and PD pathogenesis is backed up by neurotoxin-induced animal models (6-hydroxydopamine (6-OHDA), rotenone, and 1-methyl-4-phenyl-1,2,3,6-tetrahydropyridine (MPTP)), which result in ROS generation and gradual loss of nigrostriatal dopaminergic system.^73-76^

**Figure 1:**
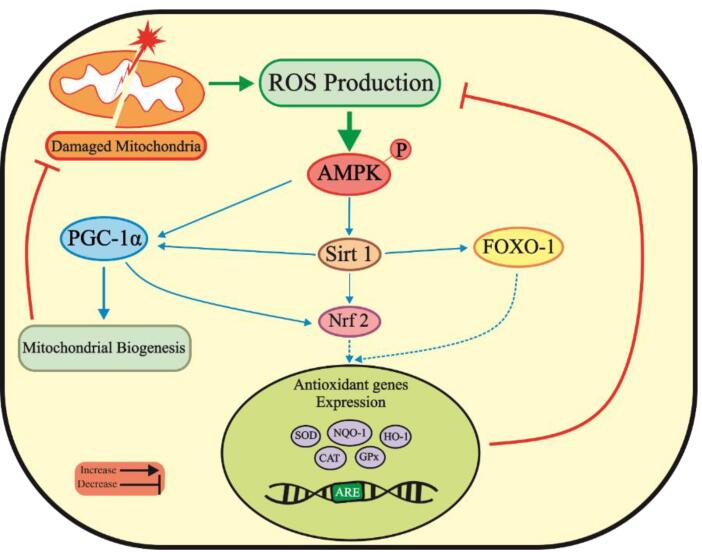
Activated AMPK suppresses oxidative stress pathways related to PD by activating PGC-1α and Sirt 1 pathways, resulting in increased antioxidant gene expression and inhibition of mitochondrial damages. ROS, Reactive oxygen species; AMPK, AMP-activated protein kinase; FOXO-1, Forkhead box class O family member proteins-1; Sirt 1, Sirtuin 1; PGC-1α, Peroxisome Proliferator-activated receptor-gamma coactivator-1; Nrf2, Nuclear factor E2-related factor 2; SOD, Superoxide dismutase; NQO-1, NAD(P)H quinone dehydrogenase 1; HO-1, Heme oxygenase-1; CAT, Catalase; GPx, Glutathione peroxidase

 Aberrant production of ROS and imbalanced redox status activate AMPK to maintain redox homeostasis. AMPK promotes the expression of antioxidant enzymes such as glutathione peroxidase (GPx), superoxide dismutase (SOD), and catalase (CAT) to mitigate ROS generation by activating Sirt1/PGC-1α/FOXO-1 pathway (Figure 1). However, pharmacological or genetic inactivation of AMPK leads to elevated mitochondrial ROS levels, promoting cytotoxicity.^69^ Nuclear factor E2-related factor 2 (Nrf2) maintains redox balance and protects cells from oxidative damage. Nrf2 is usually kept in the cytoplasm during stress-free conditions, but it translocates to the nucleus on exposure to oxidative stress. Once bound to the antioxidant response element, it activates the expression of several antioxidative enzymes, including heme oxygenase-1 (HO-1), SOD, and GPx, which help to detoxify free radicals. Through phosphorylation, AMPK also enhances Nrf2 nuclear translocation, thus reducing ROS levels and inhibiting oxidative stress.^77,78^ Therefore, activating the AMPK pathway may serve as a therapeutic approach for inhibiting oxidative stress in PD.

## Effect of AMPK on macroautophagy

 Autophagy is a process that transfers waste products, cellular components, and large molecules to the lysosome for decomposition and ingestion.^24^ Autophagy disturbance is one of the etiologies of PD, leading to α-syn accumulation in the brain.^79^ Moreover, deleting essential genes involved in autophagy, such as autophagy-related gene-7 (*ATG-7)*, can induce neurodegeneration similar to PD in mice.^80^ A recent study has shown that tricin, a natural flavonoid, can improve autophagy and ATG-7-dependent clearance of α-syn via an AMPK/mTOR pathway.^81^

 There are three main ways to remove α-syn from neurons: the ubiquitin-proteasome system (UPS), chaperone-mediated autophagy (CMA), and macroautophagy.^82^ Removing α-syn oligomers requires macroautophagy-mediated degradation because UPS and CMA are ineffective. To accomplish this, autophagosomes are formed to separate cytoplasmic components and carry them to lysosomes.^83,84^ In both PD patients and animal models, macroautophagy is stimulated by transcription factor EB (TFEB), which mediates lysosomal biogenesis and macroautophagy development due to increased α-syn levels.^85,86^ In the PD mice model, overexpression of α-syn causes macroautophagy dysfunction and increases dopaminergic neuron degeneration in SNpc and movement disorders. These defects can be improved by overexpression of TFEB or Beclin-1 (another autophagy regulator), suggesting that macroautophagy regulation can be helpful in the PD to reduce α-syn accumulation and neuronal damage.^86,87^

 Autophagy initiation is mainly driven by ULK-1, while the inhibition of ATG-13 phosphorylation by mTORC-1 leads to a decrease in the ULK-1 complex activity, ultimately suppressing autophagy.^88,89^ ULK-1 factor initiates the formation and maturation of autophagosomes through the Beclin-1 phosphorylation.^90^ Evidence suggests that AMPK boosts autophagic degradation by activating ULK-1 through phosphorylation and inhibiting mTORC1 and blocking its inhibitory effect on ULK-1.^91^ Moreover, AMPK promotes lysosomal biogenesis by increasing the activity of TFEB^92^ and improving the transcription of proteins required for macroautophagy by FOXO-3.^93^ Preclinical studies indicate that autophagy-promoting agents can improve α-syn clearance and provide neuroprotection.^94^ Metformin has been shown to stimulate autophagy and protect nigrostriatal neurons in PD models by activating the AMPK/FOXO-3 pathway.^53,95,96^ Moreover, resveratrol exhibits neuroprotective properties in PD models by inducing autophagy via AMPK activation and mTOR inhibition.^97,98^ Therefore, AMPK-dependent stimulation of autophagy may hold promising potential for developing new therapeutic strategies in PD.

## Effect of AMPK on genetic PD

 Genetic PD is rare; however, several types are identified that account for almost 30% of familial cases.^99^ Genetic mutations in *LRRK2*, *PARK2*, *PARK7*, *PINK1*, or the *SNCA* gene can lead to familial cases of PD. Accumulating studies show that mutations in *SNCA*, *GBA*, and *LRRK2* genes result in overexpression of α-syn and increased secretion of pro-inflammatory cytokines, leading to the development of motor dysfunction.^92,100-102^

*PARK7* (also known as DJ-1) is the gene responsible for the expression of DJ-1 protein, and its mutation causes genetic form and early onset of PD.^103^ A critical function of DJ-1 is nuclear communication with mitochondria.^104^ The wild-type DJ-1 enzyme prevents glycolysis metabolite damage in cells metabolizing carbohydrates.^105^ It protects cells from oxidative stress-induced cytotoxicity by enhancing Nrf2 transcriptional activity and preventing Nrf2 inactivation.^106,107^ Moreover, DJ-1 is one of the influential factors in cellular signals, including transcription of tyrosine hydroxylase, dopamine receptor, and p53 signaling pathway.^104^ *PINK1* is also transcriptionally up-regulated by Nrf2, which shields dopamine neurons from neurotoxicity induced by oxidative stress.^106,107^ AMPK can enhance Nrf2 nuclear translocation through phosphorylation and inhibiting oxidative stress.^77,78^

*PARKIN*, *PINK1*, *LRRK2, *and* PARK7* genetic mutations cause mitochondrial morphology and function abnormalities.^108^ Point mutations in the *PARK7* (NM_007262.5) gene include p.Leu166Pro(c.497T > C), p.Ala104Thr (c.310G > A), p.Met26Ile(c.78G > A), p.Asp149Ala (c.446A > C), p.Glu64Asp(c.192G > C), p.Leu10Pro(c.29T > C), and p.Pro158del(c.471_473del).^109^ Activation of AMPK by adaptor protein phosphotyrosine interacting with PH domain and leucine zipper (APPL)-1, an endosomal adapter protein, can protect against the p.Leu166Pro(c.497T > C) mutation of the *PARK7* gene.^110^

## Effect of AMPK on inflammation

 Both preclinical and clinical PD studies have proved that the onset and progression of PD involve neuroinflammation and immune dysfunction.^111^ The causes of inflammation in PD include exposure to heavy metals, environmental toxins, bacterial and viral infections, and pesticides.^112^

 Microglia, a part of the innate immune system in the central nervous system (CNS), are categorized into M1 and M2 subtypes. The M2 phenotype has anti-inflammatory and cytoprotective properties, essential for maintaining CNS homeostasis. Upon microglia activation, the M2 subtype is transformed into the M1 subtype, known to be cytotoxic and pro-inflammatory.^113-115^ In the pathology of PD, the accumulation of α-syn and the increase of ROS in dopaminergic cells promote neuronal death, followed by the release of damage-associated molecular patterns (DAMPs) from neurons, resulting in an increase in the activity of M1 microglia in the CNS.^116^ Preclinical PD models have shown that microglial activation and secretion of pro-inflammatory cytokines, particularly IL-6 and IL-1β, precede the degeneration of dopamine neurons.^117,118^ Additionally, there is a connection between pathological α-syn accumulation and the PD brain’s heightened inflammation.^119,120^

 The blood-brain barrier becomes weaker when inflammation increases in the brain, leading to the penetration of harmful substances like ROS and NO, which cause further damage.^121^ In a 6-OHDA-induced PD model, the amount of pro-inflammatory cytokines such as IL-1, IL-6, TNF-α, and INF-γ were increased, while anti-inflammatory cytokines such as IL-10 was decreased, indicating dysregulation in the immune system and the occurrence of inflammation in the CNS.^122^ In basal condition, nuclear factor kappa B (NF-κB) is inactive, localizes in the cytoplasm, and tightly bound to an inhibitor of nuclear κB (IκB). Upon activation by DAMPs, IκB kinase (IKK) targets IκB for degradation, resulting in translocation of NF-κB to the nucleus, pro-inflammatory gene expression, and damage to dopaminergic neurons through impaired mitochondrial function and autophagy by suppressing Sirt1/FOXO-PGC-1α pathway.^123,124^

 On the other hand, nuclear receptor-related protein 1 (NURR1) controls the expression of genes essential for the survival of dopaminergic neurons and has the potential to inhibit NF-κB activity when activated.^125^ Nrf2 transcription factor not only boosts antioxidative defense but also plays a critical role in regulating inflammation and has been substantiated to obstruct inflammatory responses prompted by inflammatory factors. Typically, Nrf2 is expressed at high levels in glial cells, and its activation reduces neuroinflammation.^121^ The survival of dopaminergic neurons is influenced by Nrf2 and NF-κB, which behave as antagonistic transcription factors. Nrf2 negates NF-κB signaling, while NF-κB silences Nrf2 target genes and deprives it of necessary co-transcription factors. However, a lack of Nrf2 results in an increase in NF-κB levels through proteasome-mediated IκB degradation. Therefore, activation of the Nrf2 pathway can alleviate PD symptoms by reducing cellular damage from oxidative stress and neuroinflammation, as well as improving mitochondrial function.^126,127^

**Figure 2:**
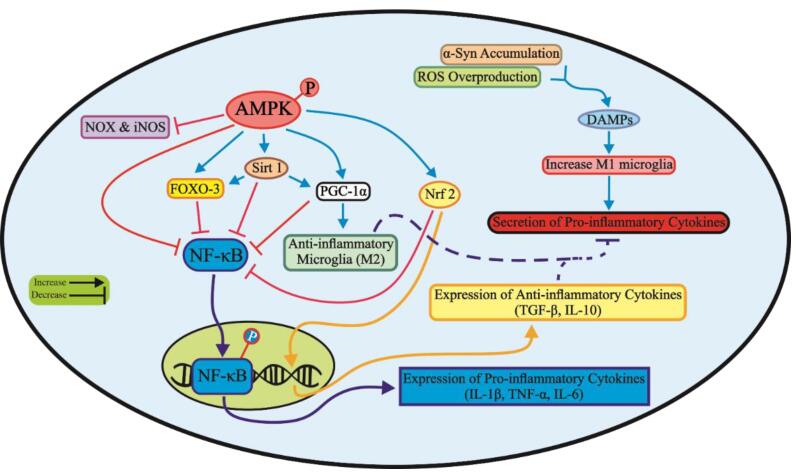
The phosphorylation of AMPK targets multiple signaling pathways to inhibit neuroinflammation responses related to PD. This results in the suppression of NF-κB translocation to the nucleus and expression of pro-inflammatory cytokines while enhancing the expression of anti-inflammatory cytokines. AMPK, AMP-activated protein kinase; NOX, NADPH oxidase; iNOS, Induciblenitric oxide synthase; FOXO-3, Forkhead box class O family member proteins-3; Sirt 1, Sirtuin 1; PGC-1α, Peroxisome proliferator-activated receptor-gamma coactivator-1; Nrf2, Nuclear factor E2-related factor 2; NF-κB, Nuclear factor kappa B; IL, Interleukin; TNF-α, Tumor necrosis factor-α; DAMP, Damage-associated molecular patterns; ROS, Reactive oxygen species; α-Syn: α-Synuclein

 Evidence suggests that chronic inflammation leads to a gradual decrease in AMPK function,^128^ while an increase in AMPK activity encourages microglial anti-inflammatory M2 polarization.^129^ Furthermore, AMPK suppresses NF-κB activation in the brain to inhibit inflammatory responses.^130,131^ In an MPTP-induced PD model, liraglutide was shown to modulate the AMPK/NF-κB pathway, leading to improvements in PD-related motor symptoms, rescue of dopaminergic neurons, and diminished activated microglia in the SN.^132^ Another pathway by which AMPK regulates inflammation is sirtuin1 (Sirt1). Indole-3-carbinol was reported to activate the AMPK/Sirt1 pathway and reduce nervous system inflammation in PD model mice.^133^ Moreover, AMPK reduces inflammation by inhibiting NOX-mediated ROS production and decreasing nitric oxide synthase (iNOS)-mediated nitric oxide (NO) production.^134–137^ AMPK also acts as a cofactor for Sirt1 activity and Sirt1 activation protects dopaminergic neurons through inhibiting iNOS, p53, and NF-κB expression, and increasing FOXO-3/PGC-1α pathway.^138–141^ The next target of AMPK in nerve cells to deal with neuroinflammation is activation of Nrf2.^142^ As shown in Figure 2, AMPK activation through modulation of several pathways can protect dopaminergic neurons from inflammation.

## Effect of AMPK on cell survival and apoptosis

 In PD, the activation of the intrinsic apoptotic pathway induces the death of dopaminergic neurons in the SNpc. Many studies suggest that PD is connected with mitochondrial-mediated apoptosis, leading to an increase in pro-apoptotic factors like BAX and cytochrome c, caspase-9, and caspase-3, and a decrease in anti-apoptotic factors such as Bcl-2 and Bcl-XL. As mentioned, PD is associated with a chain of events that drive cells toward apoptosis, including genetic mutation, accumulation of α-syn, neuroinflammation, ROS production, and mitochondrial dysfunction.^48,143-145^ Besides, genetic mutation of PD-related genes, namely *Parkin*, *LRRK2*, *PINK1*, and *PARK7*, contribute to mitochondrial impairment and apoptosis.^99,143^

 AMPK plays a dual role in regulating cell death and survival, depending on the type of stress and cells, and duration of exposure.^146-148^ Some studies have shown that the activation of AMPK for a prolonged duration can activate c-Jun N-terminal protein kinase (JNK), leading to apoptosis in liver cells and pancreas beta cells.^149,150^ However, another study showed that activation of AMPK inhibited dexamethasone-induced apoptosis in thymocytes.^151^ Conversely, some studies suggest that the activation of AMPK-related pathways could prevent the apoptosis pathway, particularly in neurons, by correcting mitochondrial abnormalities. Furthermore, 5-aminoimidazole-4-carboxamide ribonucleoside (AICAR) triggers AMPK activation that prevents apoptosis while inhibiting AMPK activity induces cell apoptosis.^152-156^ Additionally, an in vitro study demonstrated that disruption of the AMPK/Sirt1 signaling pathway by sevoflurane caused an increase in the apoptosis rate in neural cells while promoting AMPK level can improve apoptosis.^157^ In the intracerebral hemorrhage model, activating the αVβ5/AMPK pathway by Irisin, a myokine, inhibited apoptosis in the brain.^158^ In an MPTP-induced PD model, activation of the AMPK/MAPK pathway by osmotin administration reduced α-syn and apoptosis-related proteins.^159^ Besides, treatment with the AMPK agonist, GSK621, attenuated mitochondrial dysfunction and apoptotic neuronal death in the SNpc in the MPTP-induced PD mice model by activating the AMPK/GSK-3β/PP2A.^160^ Therefore, the regulation of apoptosis by AMPK is a controversial topic that requires more study.

 AMPK activation provides a significant neuroprotective effect and enhances cell survival against several cytotoxic agents. The mechanisms that AMPK activation may use to regulate PD-related pathology were summarized in this review (Table 1).

**Table 1:** The AMPK downstream pathways to protect dopaminergic neurons.

**Signaling pathways**	**Outcome**	**References**
AMPK/mTORC-1 AMPK/mTOR/ULK-1 AMPK/Sirt1/PGC-1α	Increases Autophagy Increases Mitophagy	^161-163^
		^164,165^
AMPK/Nrf2 AMPK/Sirt1/PGC-1α AMPK/Sirt1/Nrf2 AMPK/Nrf2/TXNIP AMPK/Sirt1/FOXO-1	Reduces Oxidative Stress	^130,133,164-167^
AMPK/PGC-1α/NF-κB AMPK/Sirt1/FOXO-3 AMPK/Sirt1/NF-κB AMPK/AKT/NF-κB AMPK/Nrf2/TXNIP	Reduces Inflammation	^130,133,166,167^
AMPK/Akt/mTOR AMPK/Sirt1/NF-κB AMPK/FOXO-3 AMPK/Sirt1/mTOR AMPK/MAPK/mTOR AMPK/GSK-3β/PP2A	Inhibits apoptosis	^95,133,161-163,168,169^

## Conclusion

**Figure 3:**
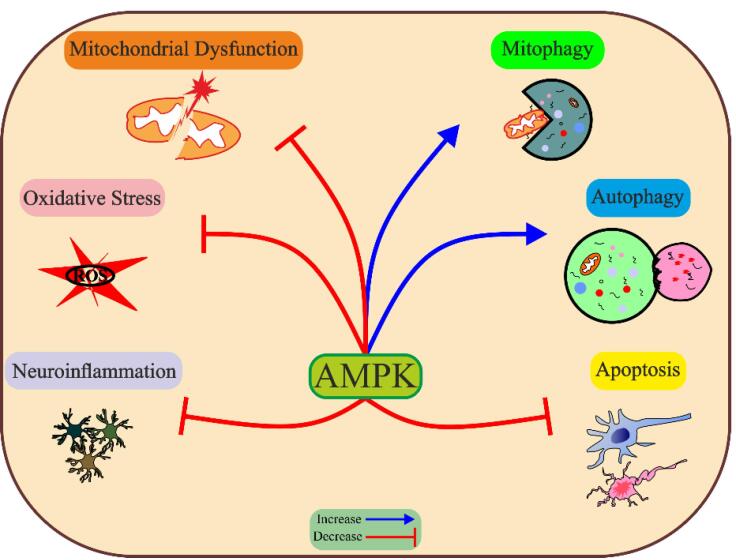
Protective effect of AMPK against Parkinson’s disease etiologies. Activated AMPK target Parkinson’s disease-related etiology by decreasing neuroinflammation, oxidative stress and apoptosis, and improving mitochondrial function, mitophagy, and autophagy. ROS, reactive oxygen species

 The pathophysiology of PD is complex and multifactorial, involving abnormalities in mitochondrial function and morphology, impaired energy metabolism, genetic mutation, aggregation of α-syn, resulting in loss of dopaminergic neurons. AMPK can regulate multiple biological functions, including mitochondrial homeostasis, mitophagy, autophagy, oxidative stress, inflammation, and apoptosis, by which effectively prevents PD-related pathology (Figure 3). To treat PD effectively, conducting additional preclinical research is necessary to gain a better understanding of the potential benefits and drawbacks of AMPK activation. This will help identify specific downstream pathways of AMPK and avoid activating any detrimental pathways.

## Acknowledgments

 The authors would like to appreciate Dr. Mahnaz Motayar and Mr. Habib Zakerani for their kind support of this study. This article aligns with Seyed Zanyar Athari’s Ph.D. thesis with ethical approval no. IR.TBZMED.AEC.1401.032.

## Competing Interests

 The authors have no relevant financial or non-financial interests to disclose.

## Ethical Approval

 This article does not contain any studies with human participants or animals performed by any of the authors.

## Funding

 No funds, grants, or other support were received.
